# Supplementary material for: Dynamic network properties of the interictal brain determine whether seizures appear focal or generalised
Source: Sci Rep. 2020 Apr 27;10:7043. doi: 10.1038/s41598-020-63430-9 (PMC7184577; doi:10.1038/s41598-020-63430-9)
Supplement: Supplementary file 1 — Supplementary Information. [file 41598_2020_63430_MOESM1_ESM.pdf]

Dynamic network properties of the interictal brain determine whether seizures appear focal or generalised

Wessel Woldman[1,2,3], Helmut Schmidt[4], Eugenio Abela[5,6], Fahmida A. Chowdhury[7], Adam D. Pawley[5,6], Sharon Jewell[5,6], Mark P. Richardson(+)[1,5,6] & John R. Terry(+)[1,2,3].
(+) These authors made an equal contribution as last author.

[1]EPSRC Centre for Predictive Modelling in Healthcare, University of Exeter, United Kingdom. [2]Centre for Biomedical Modelling and Analysis, University of Exeter, United Kingdom. [3]Centre for Systems Modelling and Quantitative Biomedicine, University of Birmingham, Birmingham, United Kingdom. [4]Max Planck Institute for Human Cognitive and Brain Sciences, CBS, Leipzig, Germany. [5]Institute of Psychiatry, Psychology and Neuroscience, King's College London, United Kingdom. [6]Department of Basic and Clinical Neurosciences, King's College London, United Kingdom. [7]NIHR University College London Hospitals Biomedical Research Centre, UCL Institute of Neurology, Queen Square, London, United Kingdom.

(*): Corresponding author: Wessel Woldman PhD, Centre for Systems Modelling and Quantitative Biomedicine, University of Birmingham, Birmingham, United Kingdom, +44-7856951932, w.woldman@bham.ac.uk

Helmut Schmidt: schmidthelmut1982@gmail.com
Eugenio Abela: eugenio.abela@kcl.ac.uk
Fahmida Chowdhury: fahmidachowdhury@hotmail.com
Adam Pawley: adampawley@hotmail.com
Sharon Jewell: sharon.1.jewell@kcl.ac.uk
Mark Richardson: mark.richardson@kcl.ac.uk
John Terry: J.Terry@bham.ac.uk

Supplementary materials: Characteristics of the study participants

Table 1: Clinical and Demographic Characteristics of Study Participants

	Subjects	Female	Age in years (SD)*; range	Duration of epilepsy in years (SD); range	Number of AEDs (n)
**Controls**	38	19 (50%)	29.9 (8.6); 18-52	-	-
**IGE**	25	15 (60%)	32.5 (11.1); 20-61	19.5 (14.7); 1-58	1 (13), 2 (9), 3 (3)
**Focal**<br>**- Left**<br>**- Right**	 23<br>20	 9 (39%)<br>13 (65%)	 38.7 (16.5); 20-77<br>35.9 (13.2); 18-68	 15.4 (14.4); 2-55<br>20.4 (11.2); 2-40	 1 (9), 2 (11), 3 (3)<br>1 (6), 2 (10), 3 (4)

*Caption***:** *Data are presented as integers n, integers n (%), or mean (SD). The Mann-Whitney U test was used to compare age, duration of epilepsy and drug load between the groups (all tests failed to reject the null-hypothesis of equal medians of the groups, two-tailed, p<0.05); Fisher's exact test was used for comparing gender (all tests failed to reject the null-hypothesis, p<0.05).*

Control cohort

**Subject**	**Gender**	**Age**
1	m	30
2	m	22
3	m	24
4	m	26
5	f	29
6	f	26
7	m	30
8	f	30
9	m	28
10	f	31
11	f	19
12	f	46
13	m	27
14	m	20
15	f	18
16	m	26
17	f	30
18	m	23
19	m	22
20	m	23
21	f	30
22	f	28

23	f	37
24	m	20
25	f	28
26	f	28
27	m	27
28	m	52
29	m	37
30	f	27
31	f	27
32	f	41
33	m	37
34	f	31
35	f	24
36	m	51
37	m	30
38	f	51

Idiopathic Generalised Epilepsy Cohort

Subject	Gender	Age (years)	Duration of disease (years)	Syndrome	Type of AED	MRI	EEG: Background Slowing	EEG: Focal Slowing	EEG: focal IEDs	EEG: gen. IEDs
1	m	31	17	GTCS	CBZ	Normal	-	-	-	-
2	f	40	31	JAE	LTG, VPL	Normal	-	-	-	-
3	f	31	5	IGE	LTG, LEV	Normal	-	-	-	-
4	f	52	50	IGE	VPL, TPM	Normal	-	-	Yes	-
5	f	47	36	JAE	LEV	Normal	-	-	-	Yes
6	m	25	21	IGE	VPL	Normal	-	-	-	Yes
7	m	25	14	IGE	VPL, TPM	Normal	-	-	-	Yes
8	f	28	8	GTCS	CBZ	Normal	-	-	-	-
9	f	21	11	JAE	LTG, ETX	Normal	-	-	-	-
10	f	39	31	IGE	LEV	Normal	-	Yes	-	-
11	f	21	15	IGE	LTG	Normal	-	-	Yes	Yes
12	f	20	5	JME	LTG, LEV	Normal	-	-	Yes	Yes
13	m	25	9	IGE	VPL	Normal	-	-	-	-
14	m	50	42	CAE	VPL, LEV	Normal	-	-	-	-
15	m	45	24	CAE	VPL, LEV, LTG	Normal	-	-	-	Yes
16	m	28	20	IGE	VPL	Normal	-	-	-	Yes
17	f	28	12	IGE	LTG	Normal	-	-	Yes	-
18	f	33	18	IGE	VPL	Not available	-	-	-	-
19	f	28	12	IGE	TPM	Not available	-	-	-	Yes
20	m	27	1	IGE	VPL	Normal	-	-	-	Yes
21	m	27	10	IGE	CBZ, LEV, ZNS	Normal	-	-	-	Yes
22	f	23	10	IGE	VPL, LTG	Normal	-	-	-	Yes
23	m	20	3	IGE	TPM	Normal	-	-	-	Yes
24	f	38	24	IGE	LTG, LEV	Not available	-	-	Yes	Yes
25	f	61	58	IGE	LEV, CLB	Not available	Yes	-	Yes	Yes

Left Focal Epilepsy Cohort

Subject	Gender	Age (years)	Duration of disease (years)	Syndrome	Type of AED	MRI	EEG: Background Slowing	EEG: Focal Slowing	EEG: focal IEDs	EEG: generalised IEDs
1	f	23	16	Left TLE	LEV, LTG	Normal	-	-	Yes	-
2	f	22	21	Left TLE	TGB	Normal	-	Yes	Yes	-
3	f	60	55	Left TLE	CBZ, LEV	Normal	Yes	Yes	Yes	-
4	m	21	5	Left TLE	CBZ	Left middle temporal gyrus focal cortical dysplasia	-		-	-
5	f	24	9	Left TLE	CBZ	Left DNET	-	-	Yes	-
6	m	77	12	Left TLE	LTG	Left MTS	-	-	Yes	-
7	f	57	14	Left frontal	CBZ, LTG ,LEV	Left frontal encephalomalacia	-	Yes	Yes	-
8	m	50	48	Left focal	CBZ	Normal	-	-	Yes	-
9	f	44	3	Left TLE	LEV	Left MTS	-	-	Yes	-
10	f	31	4	Left frontal	LTG		-	-	Yes	-
11	m	20	4	Left frontal	LTG, LEV	Marginal volume loss of central white matter	-	Yes	Yes	-
12	m	60	2	Left TLE	ZNS	Normal	-	Yes	-	-
13	m	46	11	Left extratemporal	CBZ, ZNS	Normal	-	Yes	Yes	-
14	m	25	2	Left TLE	VPL	Normal	-	-	Yes	-
15	m	20	5	Left TLE	CBZ, LEV	Left MTS	-	-	Yes	-
16	m	25	5	Left medial-parietal	CBZ, LAC	Normal	-	-	Yes	-
17	m	44	21	Left TLE	CBZ, VPL	Left DNET	-	Yes	Yes	-
18	m	46	8	Left TLE	CBZ, LTG	Left MTS	-	Yes	Yes	-
19	m	32	22	Left TLE	LEV, LTG	Left MTS	-	Yes	Yes	-
20	m	36	7	Left TLE	CBZ, LEV	Left MTS	-	-	Yes	-

21	f	63	21	Left TLE	CBZ, LTG, PGB	Left MTS	-	Yes	Yes	-
22	m	38	37	Left TLE	CBZ, LEV, VPL	Left MTS	-	-	Yes	-
23	f	26	22	Left TLE	CBZ, LEV	Left MTS	-	Yes	Yes	Yes

Right Focal Epilepsy Cohort

Subject	Gender	Age (years)	Duration of disease (years)	Syndrome	Type of AED	MRI	EEG: Background Slowing	EEG: Focal Slowing	EEG: focal IEDs	EEG: generalised IEDs
1	f	47	35	Right TLE	CBZ, LTG	Right MTS	-	Yes	Yes	-
2	m	26	19	Right TLE	CBZ, LEV, CLB	Right MTS	-	-	Yes	-
3	f	20	10	Right TLE	CBZ, TPM	Right MTS	-	Yes	Yes	-
4	m	31	20	Right TLE	CBZ, LEV	Right MTS	-	Yes	Yes	-
5	f	18	3	Right TLE	CBZ, TPM	Right MTS	-	-	Yes	-
6	f	21	2	Right TLE	LEV, LTG	Right MTS	-	Yes	Yes	-
7	f	39	30	Right TLE	CBZ, TPM	Right MTS	-	Yes	Yes	-
8	f	24	13	Right TLE	OXC	Right MTS	-	Yes	Yes	-
9	m	68	40	Right TLE	VPL, PHT	Right encephalomalacia of fusiform gyrus	-	-	Yes	-
10	f	37	17	Right focal	CBZ, TPM	Normal	-	-	-	-
11	f	41	28	Right frontal	LEV, PHT	Right gliosis and hemosiderin deposit after superior frontal gyrus cavernoma resection	-	Yes	Yes	Yes
12	f	26	25	Right temporal	LTG	Normal	Yes	-	-	-
13	f	52	38	Right TLE	PHT, TPM, LAC	Right small focal cortical abnormality in the inferomedial temporal lobe	-	-	Yes	-
14	f	38	13	Right TLE	LTG	Right malformation of	-	-	Yes	-

						brain development in the temporal occipital area and possible MTS in addition				
15	f	45	33	Right frontal	VPL, LAC	Right frontal meningeoma, status after resection of left temple meningeoma	-	Yes	Yes	-
16	m	22	16	Right frontal	LTG	Normal	-	-	Yes	-
17	f	48	21	Right TLE	LEV, VPL, TPM	Right DNET	-	-	Yes	-
18	m	51	17	Right TLE	VPL, LTG, LAC	Normal	-	-	Yes	-
19	m	33	23	Right TLE	VPL	Right MTS	-	Yes	Yes	-
20	m	31	5	Right TLE	TPM	Right temporal lobe malformation with dysplastic malrotate hippocampus and diffuse nodular heterotopia	-	-	Yes	-

Abbreviations: m: male; f: female; GTCS: generalised tonic-clonic seizures only; CAE: childhood absence epilepsy; JAE: juvenile absence epilepsy; IGE: idiopathic generalised epilepsy (unclassified); TLE: temporal lobe epilepsy; MTS: mesial temporal sclerosis; DNET: dysembryobastic neuroepithelial tumor; IED: interictal epileptiform discharge; CBZ: carbamazepine; CLB: clobazam; ETX: ethosuximide; LAC: Lacosamide; LEV: levetiracetam; LTG: lamotrigine; OXC: Oxcarbazepine;

PGB: Pregabalin; PHT: Phenytoin; TGB: Tiagabine; TPM: toparimate; VPL: valproate; ZNS: zonisamide.

Table: Group Comparisons

	**Sex**	**Age**	**Duration**	**Number of AEDs**
**Controls – IGE**	0.4341	0.4991	-	-
**Controls – Left Focal**	0.4053	0.1065	-	-
**Controls – Right Focal**	0.4398	0.1043	-	-
**IGE – Left Focal**	0.1482	0.4436	0.2075	0.4479
**IGE – Right Focal**	0.9999	0.3686	0.4786	0.1533
**Left Focal – Right Focal**	0.1292	0.7607	0.0902	0.4643

*Caption***:** *The Mann-Whitney U test (two-tailed, $p<0.05$) was used to compare age, duration of epilepsy and drug load between the groups. Fisher's exact test ($p<0.05$) was used for comparing sex. All tests failed to reject the null-hypothesis of equal medians of the groups.*

Figure S1: Exemplar Network Structures & Distributions

Figure S1A: Example network structure for a control subject (subject 1 in Control table above)

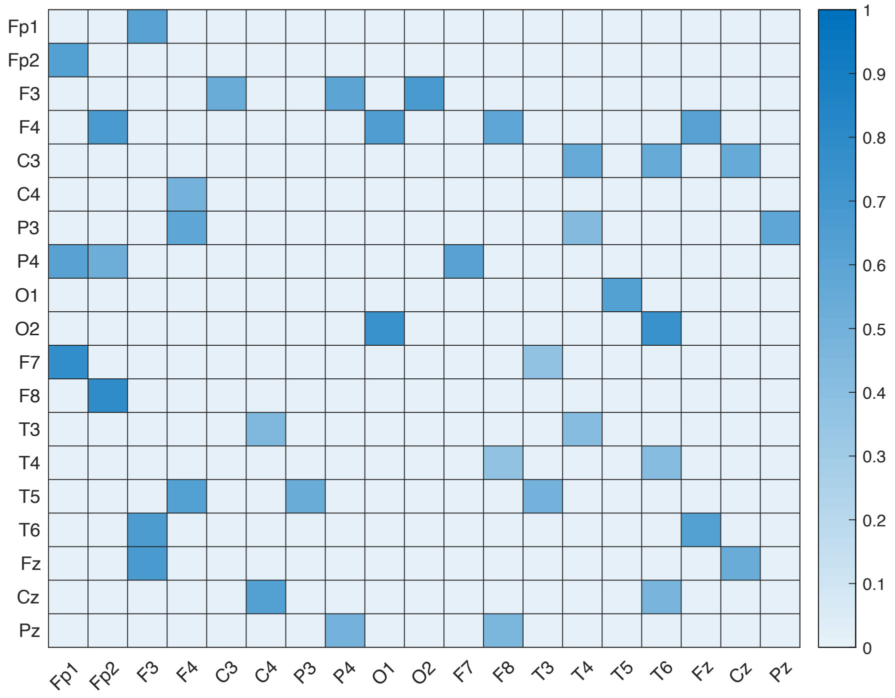

Figure S1B: Example network structure from subject with generalised epilepsy (subject 1 in IGE table above)

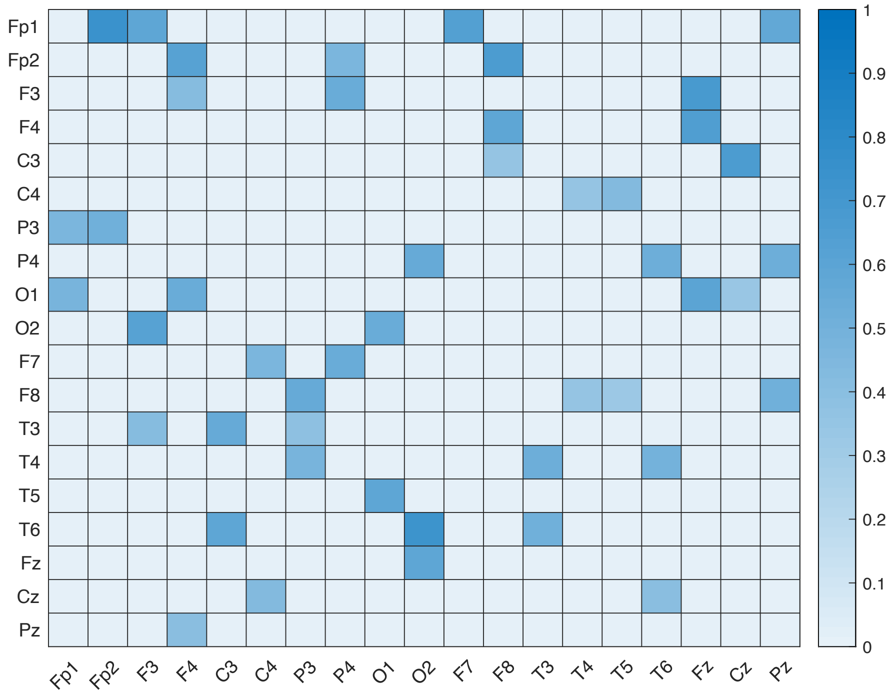

Figure S1C: Example network structure from subject with left TLE (subject 1 in Left Focal table above)

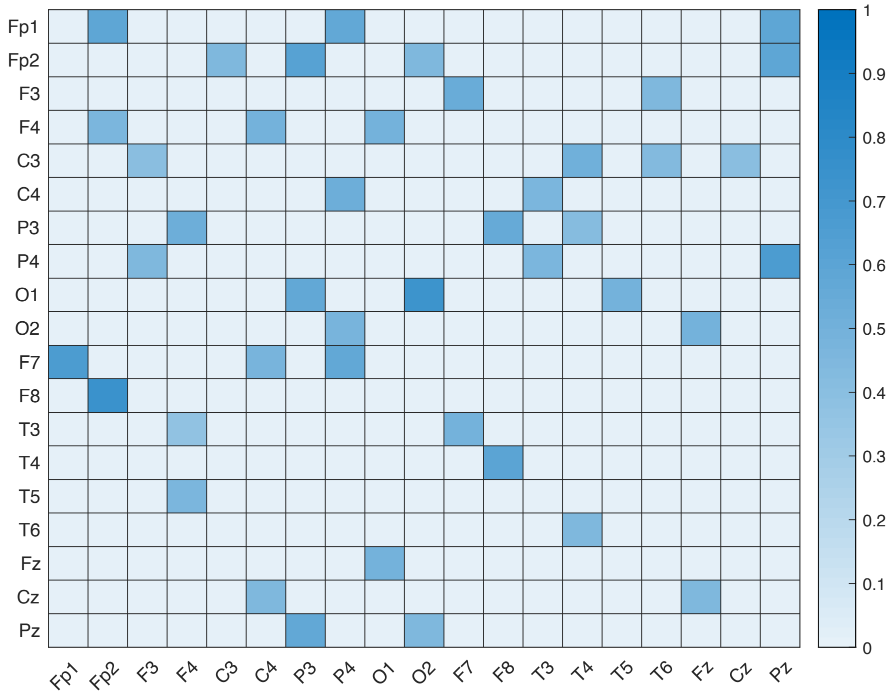

Figure S1D: Example network structure from subject with right TLE (subject 1 in Right Focal table above)

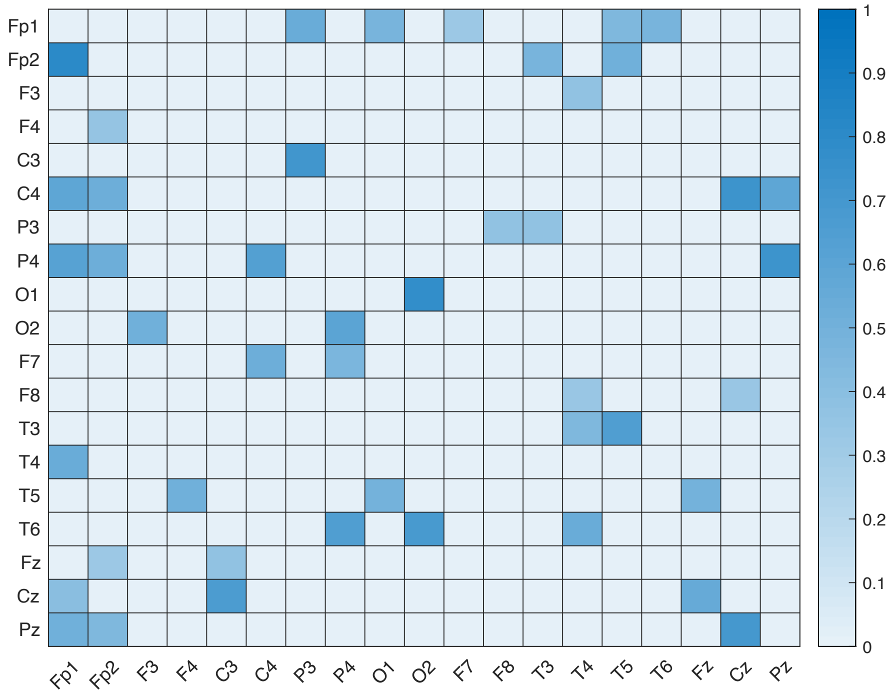

Figure S1E: Mean and standard deviation for the functional networks from the control cohort

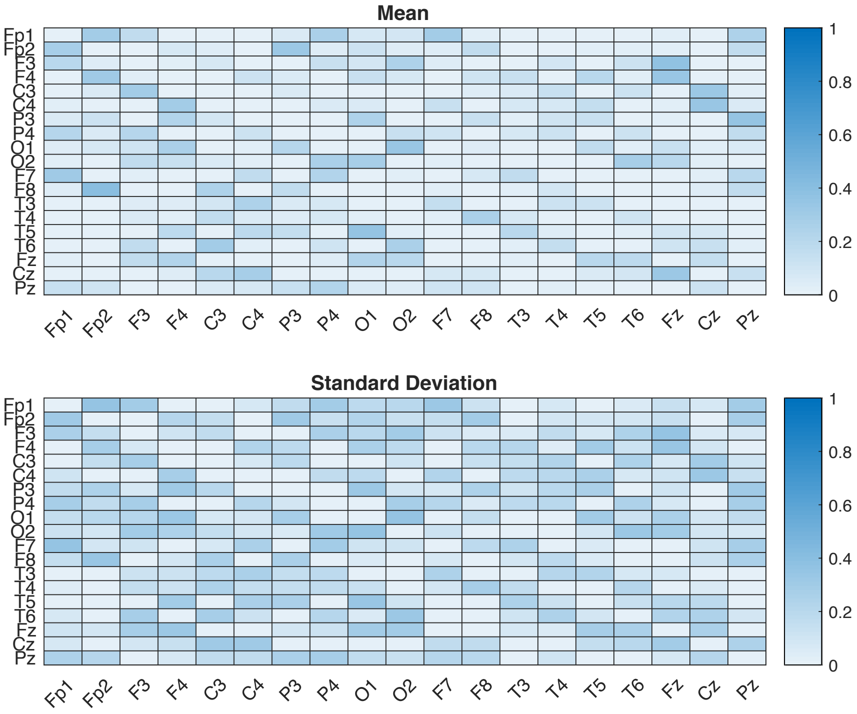

Figure S1F: Mean and standard deviation for the functional networks from the epilepsy cohort

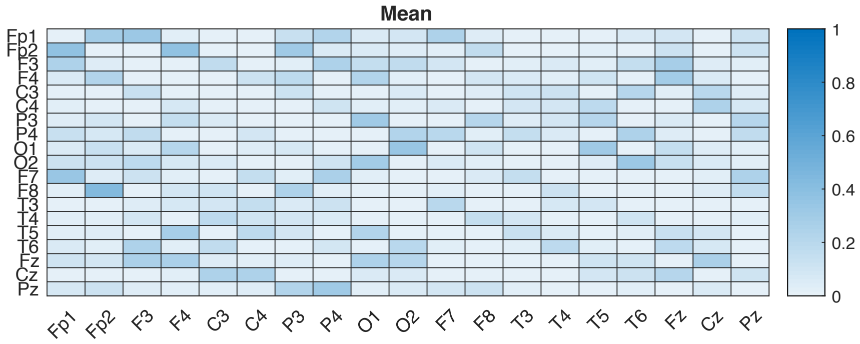
Mean
Fp1
Fp2
F3
F4
C3
C4
P3
P4
O1
O2
F7
F8
T3
T4
T5
T6
Fz
Cz
Pz
0
0.2
0.4
0.6
0.8
1

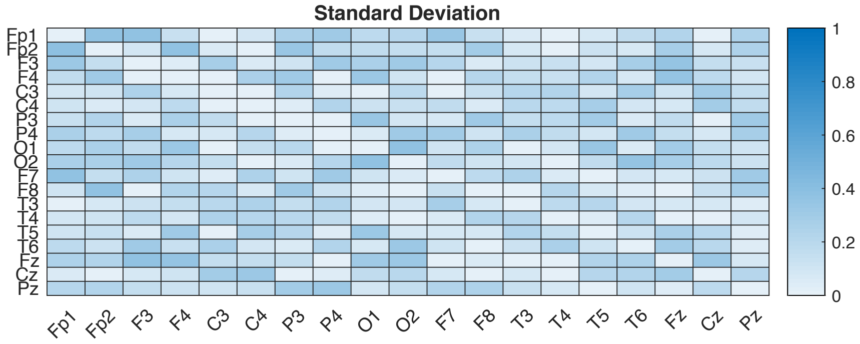
Standard Deviation
Fp1
Fp2
F3
F4
C3
C4
P3
P4
O1
O2
F7
F8
T3
T4
T5
T6
Fz
Cz
Pz
0
0.2
0.4
0.6
0.8
1

Table 2: Multiple Comparisons
Benjamini-Hochberg-procedure with 3420 univariate surrogates (for each individual candidate network) and an FDR of 5%:

Table 2.1a: Critical Coupling
Benjamini-Hochberg Procedure
Kruskal Wallis: p=0.00003

Group-comparison	p-value (MWU)	BC: x3
Con – IGE	0.0011 (U = 708)	0.0032
Con – Focal	<0.0001 (U = 1253)	0.0001
Focal – IGE	0.0969 (U = 407)	0.2908

Table 2.2: Onset Index and Participation Index
Benjamini-Hochberg Procedure

Group-comparison	p-value (MWU)
OI: Focal-IGE	p = 0.0079 (U = 328)
PI: Focal-IGE	p = 0.0382 (U = 374)

Table 2.3: Significantly Different Regions in Left Focal, Right Focal, and IGE
Benjamini-Hochberg Procedure
Left Focal

Onset Index		Participation Index	
**Channel**	**p-value (BC: 19x3)**	**Channel**	**p-value (BC: 19x3)**
F7	0.030 (U = 204)	Fp2	0.0170 (U = 194)
C3	0.027 (U = 202)	F3	0.0170 (U = 194)
T3	0.049 (U = 213)	T5	0.0095 (U = 184)

Right Focal

		Participation Index	
		Channel	p-value (BC: 19x3)
		Fp2	0.0249 (U = 165)

In general, the trends and found differences are very similar and qualitatively identical. However, the observed effect in the right focal cohort for the Onset Index is rejected after the BH-procedure, which is unsurprising since this effect was close to the cut-off value (0.048).
